# A 2,5-Dihydroxybenzoic Acid–Gelatin Conjugate: The Synthesis, Antiviral Activity and Mechanism of Antiviral Action Against Two Alphaherpesviruses

**DOI:** 10.3390/v7102878

**Published:** 2015-10-15

**Authors:** Alexander Lisov, Veronika Vrublevskaya, Zoy Lisova, Alexey Leontievsky, Oleg Morenkov

**Affiliations:** 1 Skryabin Institute of Biochemistry and Physiology of Microorganisms, Russian Academy of Sciences, Prospekt Nauki 5, Pushchino, Moscow Region 142290, Russia; zoyapozhid@rambler.ru (Z.L.); leont@ibpm.pushchino.ru (A.L.); 2 Institute of Cell Biophysics, Russian Academy of Sciences, Pushchino, Moscow Region 142290, Russia; v_vrublevskaya@mail.ru (V.V.); morenkov_o@mail.ru (O.M.); 3 Pushchino State Institute of Life Sciences, ProspektNauki 3, Pushchino, Moscow Region 142290, Russia

**Keywords:** laccase, 2,5-dihydroxybezoic acid (2,5-DHBA), 2,5-DHBA–gelatin conjugate, pseudorabies virus, bovine herpesvirus type 1, antiviral activity, virus adsorption

## Abstract

Various natural and synthetic polyanionic polymers with different chemical structures are known to exhibit potent antiviral activity *in vitro* toward a variety of enveloped viruses and may be considered as promising therapeutic agents. A water-soluble conjugate of 2,5-dihydroxybezoic acid (2,5-DHBA) with gelatin was synthesized by laccase-catalyzed oxidation of 2,5-DHBA in the presence of gelatin, and its antiviral activity against pseudorabies virus (PRV) and bovine herpesvirus type 1 (BoHV-1), two members of the *Alphaherpesvirinae* subfamily, was studied. The conjugate produced no direct cytotoxic effect on cells, and did not inhibit cell growth at concentrations up to 1000 µg/mL. It exhibited potent antiviral activity against PRV (*IC*_50_, 1.5–15 µg/mL for different virus strains) and BoHV-1 (*IC*_50_, 0.5–0.7 µg/mL). When present during virus adsorption, the conjugate strongly inhibited the attachment of PRV and BoHV-1 to cells. The 2,5-DHBA–gelatin conjugate had no direct virucidal effect on the viruses and did not influence their penetration into cells, cell-to-cell spread, production of infectious virus particles in cells, and expression of PRV glycoproteins E and B. The results indicated that the 2,5-DHBA–gelatin conjugate strongly inhibits the adsorption of alphaherpesviruses to cells and can be a promising synthetic polymer for the development of antiviral formulations against alphaherpesvirus infections.

## 1. Introduction

At present, many efforts are made to discover new natural and synthetic antiviral compounds. It is well documented that numerous negatively charged polymers exhibit potent antiviral activity against various enveloped viruses [1,2]. Thus, sulfated polysaccharides, such as heparin, fucoidan, dextran sulfate, pentosan polysulfate, or the polyaromatic compound lignin inhibited the replication of human immunodeficiency virus (HIV), herpes simplex virus (HSV), influenza virus, human cytomegalovirus (HCMV), and vesicular stomatitis virus (VSV) [3,4,5,6,7,8]. Humic acids were shown to be active against HSV [9]. The synthetic polysaccharides dextran sulfate, sulfated chitin, and octadecylribo-oligosaccharides showed high antiviral activities [10,11,12]. Polyvinyl alcohol sulfate and its copolymer with acrylic acid demonstrated a potent inhibition of HSV, HCMV, VSV, respiratory syncytial virus, toga-, arena- and retroviruses [13]. Sulfated lignins were reported to possess strong antiviral activity against HSV-1, HSV-2, and HIV-1 [14]. Antiviral activity was exhibited by metal-containing polymers [15] and the synthetic polymer naphthalene sulfonate [16]. Lignin-like dehydrogenated polymers (DHP) obtained by oxidative polymerization of hydroxyphenyl propionic acids are strong antiviral agents; e.g., DHP of caffeic and ferulic acid exhibited strong anti-HSV-1 activity, higher than that of synthetic sulfated lignins [17]. Synthetic polyhydroxycarboxylates produced from phenolic compounds were shown to inhibit the replication of HSV, HCMV, and HIV [18,19].

The enzyme laccase (benzenediol: oxygen oxidoreductase, EC 1.10.3.2) is widely used for the oxidative polymerization of various phenolic compounds [20]. It contains four atoms of copper per molecule and belongs to the group of multicopper oxidases. The enzyme was found in fungi, plants, bacteria, lichens, and insects [21,22,23,24,25]. Laccase catalyzes the oxidation of a wide range of substrates by oxygen, which reduces to water during the reaction. Various aromatic compounds, primarily phenolic or aromatic amines, can be used as laccase substrates. Laccase oxidizes substrates to form intermediate radicals or quinones, which interact with one another or with other compounds to generate various products. This property of the enzyme is used in organic synthesis, including the synthesis of polymers, and in polymer grafting [20]. Laccase is used to prepare DHP and copolymer conjugates of phenols with various compounds. Oxidation of phenols such as 2,6-dimethoxyphenol, catechol, and ferulic acid by laccase results in water-insoluble polymers [26,27,28]. Laccase can mediate the synthesis of water-soluble copolymers by copolymerization of phenolic compounds with acrylic acids [29]. The enzyme is capable of forming cross-links in protein molecules, especially in the presence of phenols [30]. In the presence of the enzyme, gelatin-catechin conjugates with antioxidant properties are formed [31]. The gallic acid-gelatin conjugate prepared using laccase inhibited enzymes involved in the pathogenesis of Alzheimer’s disease and showed an anticancer activity [32].

The alphaherpesvirus subfamily of herpesviruses consists of closely related members, including HSV-1 and HSV-2, pseudorabies virus, varicella-zoster virus, bovine herpesvirus type 1, equine herpesvirus type 1, and some other viruses that cause serious diseases of humans and animals. Various natural and synthetic polymers were shown to possess antiviral activity against a number of alphaherpesviruses and to impair mainly the entry of alphaherpesviruses into the cells since polymers are incapable of penetrating the cell interior (for review, see [1]). Human and animal members of the alphaherpesvirus subfamily have common mechanisms of the entry into target cells. Infection with alphaherpesviruses involves the attachment of a virus to the cell surface followed by the fusion of the virus envelope with the cell plasma membrane and, as a result, the entry of the nucleocapsid into the cytoplasm [33]. The attachment of alphaherpesviruses to target cells is mediated by the interaction of viral glycoproteins with cell surface molecules acting as virus receptors. At the initial stage, alphaherpesviruses bind via envelope glycoproteins C (gC) and B (gB) to the negatively charged heparansulfate moieties of cellular heparan sulfate proteoglycans (HSPG) present on the surface of various cells [34,35,36,37,38,39,40,41]. With a few exceptions that include varicella zoster virus, the initial attachment to cells enables alphaherpesvirus glycoprotein D (gD) to bind to one of the gD cellular receptors [42]. The binding of gD to its receptors leads to conformational changes in gD and subsequent activation of a multiglycoprotein complex involving glycoproteins gB, gD, gH and gL, which triggers the viral fusion with the cell membrane [43].

In this report, we describe the synthesis of a conjugate of 2,5-dihydroxybenzoic acid (2,5-DHBA) with gelatin and its antiviral activity against two members of the *Alphaherpesvirinae* subfamily: the pseudorabies virus (PRV) and the bovine herpesvirus 1 (BoHV-1). PRV is the causative agent of Aujeszky’s disease (pseudorabies), a highly contagious, economically significant disease of pigs. The infection with PRV causes central nervous system signs and high mortality rates in young animals, and respiratory illness in older pigs [44]. BoHV-1 is associated with several diseases in cattle: infectious bovine rhinotracheitis, infectious pustularvulvovaginitis, balanoposthitis, conjunctivitis, abortion, encephalomyelitis, and mastitis, which are recognized as serious cattle diseases of economic importance [45]. We showed that the 2,5-DHBA–gelatin conjugate possesses strong antiviral activity *in vitro* against two alphaherpesviruses and that its antiviral effect is related to the inhibition of adsorption of the viruses to target cells.

## 2. Results

### 2.1. Synthesis of 2,5-DHBA–Gelatin Conjugate

**Figure 1:**
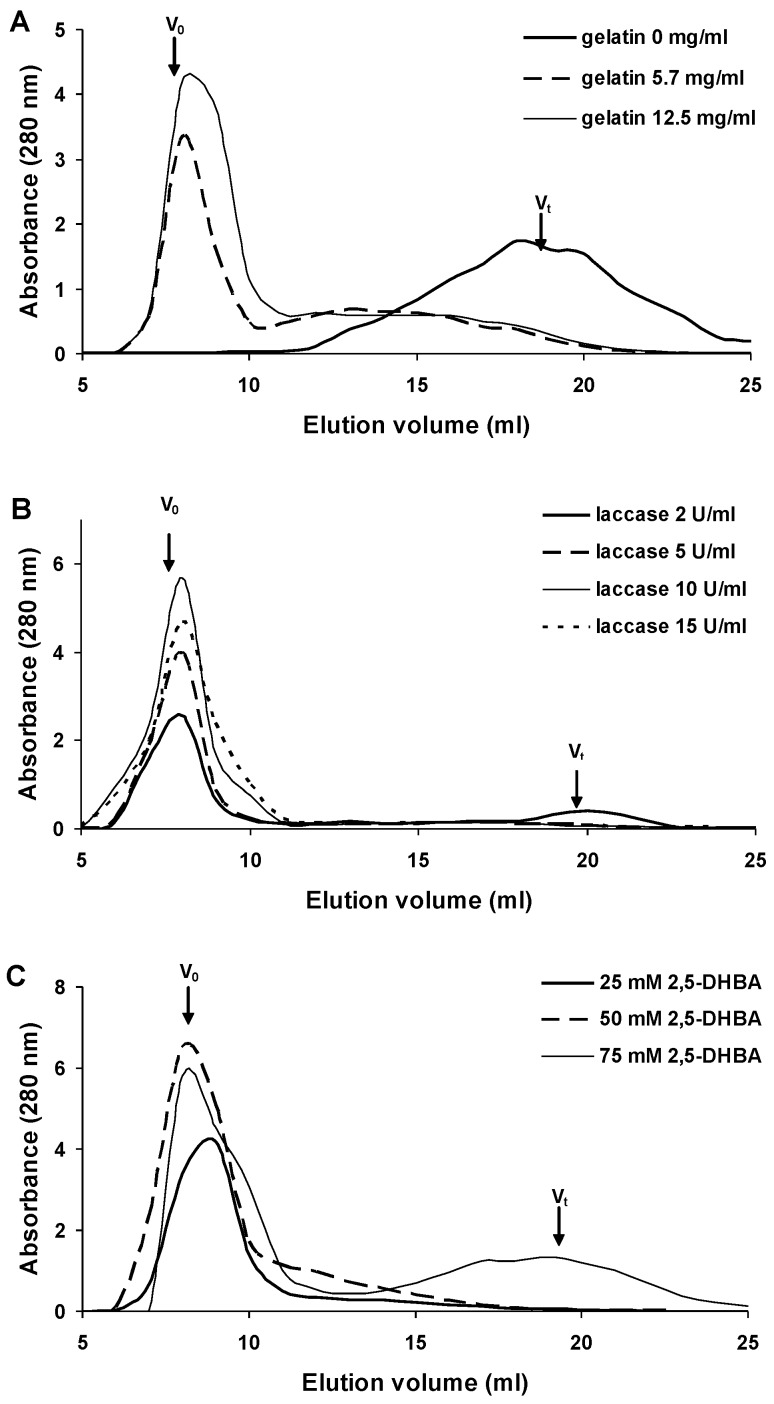
Optimization of the synthesis of the 2,5-DHBA–gelatin conjugate. Concentrations of the reactants: (**A**) gelatin—0–12.5 mg/mL, laccase—5 U/mL, 2,5-DHBA—50 mM; (**B**) laccase—2–15 U/mL, gelatin—12.5 mg/mL, 2,5-DHBA—50 mM; (**C**) 2,5-DHBA—25–75 mM, gelatin—12.5 mg/mL, laccase—10 U/mL. The data of gel filtration chromatography on Sephadex G-75 are presented. The arrows indicate the void volume (*V*_o_) and the total column volume (*V*_t_).

The 2,5-DHBA–gelatin conjugate was synthesized by laccase-catalyzed oxidation of 2,5-DHBA in the presence of gelatin. The oxidation of 2,5-DHBA at a concentration of 50 mM by laccase (5 U/mL) resulted in the formation of a brown water-insoluble precipitate. After removing the precipitate by centrifugation, the reaction mixture was light yellow in color due to the presence of low-molecular-weight products of 2,5-DHBA oxidation, eluted in the total column volume during gel filtration (Figure 1A). Thus, no water-soluble polymers formed in the reaction mixture containing 2,5-DHBA alone.

The addition of gelatin (5.7 mg/mL) to the reaction mixture resulted in the formation of a water-soluble polymer product, which was evidenced by the appearance of the peak of the polymer product on the chromatogram (Figure 1A). Increasing the concentration of gelatin to 12.5 mg/mL enhanced polymer formation. At higher gelatin concentrations, the gelation of the reaction mixture occurred. Increasing the concentration of laccase from 2 to 10 U/mL (2,5-DHBA 50 mM, gelatin 12.5 mg/mL) resulted in an enhanced formation of the polymer (Figure 1B). In the presence of laccase at a concentration of 10 U/mL, a minor amount of insoluble precipitate formed. Further increase in the amount of the enzyme lowered the concentration of the soluble polymer but significantly increased the amount of the precipitate. Reducing the amount of 2,5-DHBA to 25 mM (laccase 10 U/mL, gelatin 12.5 mg/mL) diminished polymer formation (Figure 1C). As the concentration of 2,5-DHBA was increased to 75 mM, the amount of the soluble polymer also decreased, but simultaneously low-molecular-weight products and an insoluble precipitate formed. The formation of the precipitate at increased concentrations of laccase and/or 2,5-DHBA was probably due to an excess of radicals generated by laccase. The radicals react with one another to form an insoluble polymer. The optimal concentrations of the reactants during the synthesis of 2,5-DHBA–gelatin conjugate were: 2,5-DHBA 50 mM, gelatin 12.5 mg/mL, and laccase 10 U/mL. The yield of the 2,5-DHBA–gelatin conjugate under these conditions was 70%–80%.

### 2.2. Characterization of the 2,5-DHBA–Gelatin Conjugate

**Figure 2:**
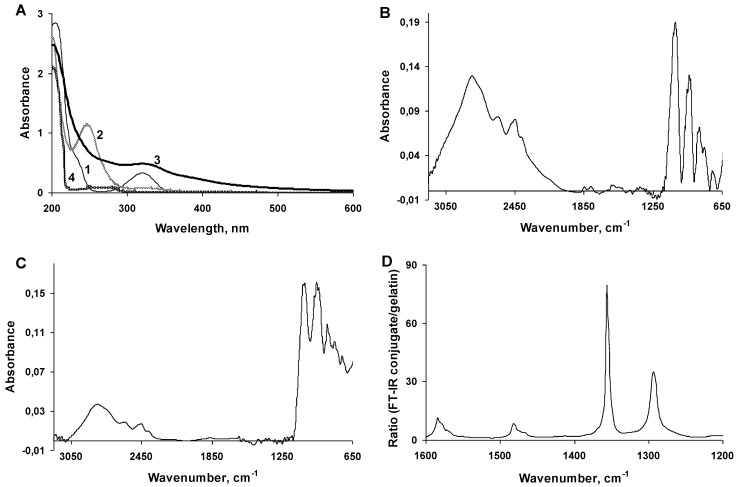
Analysis of the 2,5-DHBA–gelatin conjugate using UV-Vis (**A**) and FT-IR (**B**–**D**) spectroscopy. (**A**) Absorption spectra of 2,5-DHBA (1), laccase-oxidized 2,5-DHBA (2), 2,5-DHBA–gelatin conjugate (3), and gelatin (4); (**B**,**C**) FT-IR spectra of gelatin and the 2,5-DHBA–gelatin conjugate, respectively; (**D**) The ratio of FT-IR spectra conjugate/gelatin is given.

The conjugate resulted from the laccase-mediated polymerization of 2,5-DHBA with gelatin and the removal of low molecular compounds by the dialysis was a soluble dark brown polymer. A spectral analysis of the reactants and reaction products showed that 2,5-DHBA had an absorption maximum at 320 nm and a shoulder at 235 nm (Figure 2A). The oxidation of 2,5-DHBA by laccase without gelatin led to the formation of a product with an absorption maximum at 250 nm, which was observed for 1 h and then gradually disappeared due to the formation of the insoluble precipitate. Presumably, the oxidation of 2,5-DHBA led to the generation of quinone of 2,5-DHBA or another active intermediate, which can polymerize to form insoluble products [46]. The 2,5-DHBA–gelatin conjugate had an absorption maximum at 320 nm, probably due to the presence of 2,5-DHBA chromophore bound to gelatin, which has an absorbance peak at this wavelength (Figure 2A). FT-IR spectra of gelatin and the 2,5-DHBA–gelatin conjugate showed a close similarity (Figure 2B,C). To improve the visualization of the differences, a division of spectra copolymer/gelatin (Figure 2D) was made. In the region of 1200–1600 cm^−1^, characteristic absorption bands were present. The maximum absorption band at 1582 cm^−1^ belonged to the carboxylate anion, the 1355 cm^−1^ peak was related to vibrations of C-O in the carboxylic acid, and the peak at 1290 cm^−1^ was due to vibrations of the valence bond of C-O in phenols. The presence of the 1480 cm^−1^ peak was related to C-C bonds in aromatic rings. Thus, the FT-IR spectra indicate the presence of carboxyl groups (both free and bound), phenolic groups, and aromatic rings in the 2,5-DHBA–gelatin conjugate and hence the formation of covalent bonds between 2,5-DHBA and gelatin. The presence of substituted aromatic structures indicates that not all 2,5-DHBA functional groups (both phenolic and carboxyl) are involved in the formation of bonds with gelatin, and some of them are free.

**Figure 3:**
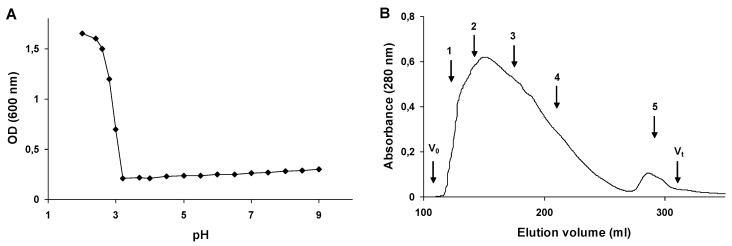
Solubility at different pH (**A**) and molecular weight determination (**B**) of the 2,5-DHBA–gelatin conjugate. (**A**) Turbidity of the conjugate solutions (optical density at 600 nm) at different pH values is presented; (**B**) The elution profile of the conjugate on HiLoad 26/60 Superdex 200 column. *V_o_*, void volume; *V_t_*, total column volume. Molecular weight markers: 1-apoferritin from horse spleen (443 kDa), 2-b-amylase from sweet potato (200 kDa), 3-alcohol dehydrogenase from yeast (150 kDa), 4-albumin, bovine serum (66 kDa), 5-carbonic anhydrase from bovine erythrocytes (29 kDa).

The 2,5-DHBA–gelatin conjugate was readily soluble in water and did not precipitate even at concentrations up to 20 mg/mL. After drying at 40 °C under vacuum or by lyophilization, the conjugate became weakly soluble in water at ambient temperature but was readily soluble in hot water (75 °C and above) and did not precipitate upon further cooling and storage. The conjugate was soluble at alkaline, neutral, and weakly acidic pH values (Figure 3A), while at pH values below 3.0 it precipitated. A gel filtration analysis of the 2,5-DHBA–gelatin conjugate revealed a major broad peak with the estimated apparent molecular weight maximum of about 175 kDa and a minor peak corresponding to a small fraction with a molecular weight of approximately 35 kDa (Figure 3B).

### 2.3. Cytotoxicity and Antiproliferative Activity of the 2,5-DHBA–Gelatin Conjugate in Vitro

The *in vitro* cytotoxicity and antiproliferative activity of the 2,5-DHBA–gelatin conjugate for BHK-21, Vero, and MDBK cells were determined using the MTT assay. The cytotoxic activity of 2,5-DHBA–gelatin was estimated by measuring the relative number of live cells after a 72-h incubation of confluent monolayers of cells in the presence of different concentrations of 2,5-DHBA–gelatin. To evaluate the antiproliferative activity of 2,5-DHBA–gelatin, cells were seeded at relatively low concentrations, incubated in the presence of different concentrations of 2,5-DHBA–gelatin, and the density of cell monolayers after a 72-h incubation of cells was determined. The results are presented in Table 1. At all concentrations used (the highest concentration 1000 µg/mL), the 2,5-DHBA–gelatin conjugate exhibited no direct cytotoxic effects on cells and did not inhibit the growth of BHK-21, Vero and MDBK cells. This implies that *CC*_50_ of the conjugate was greater than 1000 µg/mL. Free 2,5-DHBA at a concentration of 50 µg/mL showed no toxicity to all cell lines.

**Table 1:** Cytotoxic and antiproliferative activity of 2,5-dihydroxybezoic acid (DHBA)–gelatin conjugate.

Substance	Concentration, µg/mL	Cytotoxicity (%) ^a^			Antiproliferative Activity (%) ^a^		
		BHK-21	Vero	MDBK	BHK-21	Vero	MDBK
2,5-DHBA–gelatin	0	100	100	100	100	100	100
	62.5	111 ± 15	94 ± 10	n.d.	107 ± 7	90 ± 16	n.d.
	125.0	95 ± 12	99 ± 8	107 ± 12	102 ± 15	92 ± 11	96 ± 10
	500.0	103 ± 11	91 ± 13	n.d.	99 ± 11	98 ± 11	n.d.
	1000.0	95 ± 13	102 ± 11	95 ± 9	93 ± 13	92 ± 14	94 ± 12
2,5-DHBA	50.0	93 ± 15	103 ± 16	111 ± 13	n.d.	n.d.	n.d.

^a^ The results are presented as OD_550_ values (MTT assay) for wells with different concentrations of 2,5-DHBA–gelatin relative to control wells without the conjugate, expressed in percent. The OD_550_ value of control wells was taken as 100%. Each value is the mean ± SD of five to six repetitions; n.d., not determined.

### 2.4. Antiviral Activity of the 2,5-DHBA–Gelatin Conjugate

The antiviral potency of the 2,5-DHBA–gelatin conjugate was evaluated against two alphaherpesviruses: PRV (the laboratory strain Ka and the vaccine strain Bartha K61) and BoHV-1 (the virulent strain 4016). In the end-point titration assay, the viruses were titrated in a medium containing different concentrations of the conjugate. The 2,5-DHBA–gelatin conjugate showed a dose-dependent antiviral activity against two PRV strains grown on both cell cultures, BHK-21 and Vero, as well as against BoHV-1 grown on MDBK cells. Different 2,5-DHBA–gelatin preparations had the *IC*_50_ values of 1.5–5.5 µg/mL against PRV strain Ka grown on BHK-21 and Vero cells; the selectivity index was in the range of 181–667 (Table 2). For the PRV strain Bartha K61 grown on BHK-21 and Vero cells, the *IC*_50_ values were from 8.3 to 15.1 µg/mL, and the selectivity index was in the range of 66–120. The 2,5-DHBA–gelatin conjugate also demonstrated powerful inhibition of the virulent BoHV-1 strain 4016: the *IC*_50_ values were 0.5–0.7 µg/mL for different preparations of the polymer, the selectivity index was in the range of 1430–2000 (Table 2). We observed pronounced differences in the antiviral activity of the 2,5-DHBA–gelatin polymer against vaccine and laboratory PRV strains. For polymer preparation No 4, the strongest inhibition was demonstrated toward the PRV strain Ka: the *IC*_50_ values were 2.4 µg/mL and 2.8 µg/mL on BHK-21 and Vero cells, respectively; the selectivity indices were greater than 416 and 357, respectively. The 2,5-DHBA–gelatin conjugate exhibited a less potent inhibition of the PRV vaccine strain Bartha K61: the *IC*_50_ values on BHK-21 and Vero cells were 8.5 µg/mL and 10 µg/mL, the selectivity indices were greater than 117 and 99, respectively (Table 2). Free 2,5-DHBA at a concentration of 50 µg/mL exhibited no antiviral activity against PRV (Ka strain) and BoHV-1.

The observed antiviral activity of 2,5-DHBA–gelatin against two alphaherpesviruses can be attributed to the direct virucidal activity of 2,5-DHBA–gelatin or to the inhibition of one of the virus life cycle stages: attachment to cells, penetration into cells, production of infectious virus particles (uncoating, transcription and translation, assembly and release) in cells or cell-to-cell spread. The experiments aimed at elucidating the mechanism of action of the conjugate were performed using the PRV strain Ka and BoHV-1 strain 4016.

**Table 2:** Antiviral activity of 2,5-DHBA–gelatin conjugate against pseudorabies virus (PRV) and bovine herpes virus type 1 (BoHV-1).

Virus	2,5-DHBA–Gelatin Preparation Number	Antiviral Activity ^a^ (*IC_50_*, µg/mL)			Selectivity Index ^b^ (*CC_50_/IC_50_*)		
		BHK21	Vero	MDBK	BHK21	Vero	MDBK
PRV (Ka)	1	3.7 ± 0.7	1.9 ± 0.6	n.d.	>270	>526	n.d.
	2	5.5 ± 0.9	3.1 ± 0.5	n.d.	>181	>322	n.d.
	3	2.9 ± 0.5	1.5 ± 0.4	n.d.	>344	>667	n.d.
	4	2.4 ± 0.7	2.8 ± 0.6	n.d.	>416	>357	n.d.
PRV (Bartha K61)	1	12.2 ± 3.3	8.8 ± 2.8	n.d.	>82	>114	n.d.
	2	15.1 ± 4.0	8.3 ± 3.0	n.d.	>66	>120	n.d.
	4	8.5 ± 2.2	10.1 ± 2.7	n.d.	>117	>99	n.d.
BHV-1 (4016)	3	n.d.	n.d.	0.5 ± 0.1	n.d.	n.d.	>2000
	4	n.d.	n.d.	0.7 ± 0.2	n.d.	n.d.	>1430
PRV (Ka)	2,5-DHBA	>50	n.d.	n.d.	-	-	-
BoHV-1 (4016)	2,5-DHBA	n.d.	n.d.	>50	-	-	-

^a^ The antiviral activity (*IC_50_*) of 2,5-DHBA–gelatin was determined by the end-point titration assay. Each value is the mean ± SD of four to five repetitions. ^b^ *CC_50_* was taken as >1000 µg/mL. n.d., not determined.

### 2.5. Direct Virucidal Effect of the 2,5-DHBA–Gelatin Conjugate

For the evaluation of the direct influence of 2,5-DHBA–gelatin on the infectivity of PRV and BoHV-1 virions, the viruses were treated with serial dilutions of 2,5-DHBA–gelatin for 1 h at 4, 22, and 37 °C. Then, the viruses were diluted 1000-fold to reach a concentration of 2,5-DHBA–gelatin below the level that affects virus infectivity during the titration. Then virus titers were determined in virus samples. Even at high concentrations (the highest concentration 1000 µg/mL), the conjugate produced no direct virucidal effect on PRV or BoHV-1 after the incubation for 1 h at different temperatures (Table 3). This indicated that the direct virucidal effect did not contribute to the antiviral activity of 2,5-DHBA–gelatin.

**Table 3:** Virucidal activity of 2,5-DHBA–gelatin conjugate against PRV and BoHV-1.

Virus	Temperature of Incubation	Concentration of 2,5-DHBA–Gelatin (µg/mL)	Virus Titer, *TCID_50_*	Residual Infectivity (%) ^a^
PRV (Ka)	4 °C	1000	1.35 × 10^8^	101.5
		100	1.62 × 10^8^	121.8
		10	1.25 × 10^8^	92.3
		0	1.33 × 10^8^	100.0
	22 °C	1000	0.95 × 10^8^	90.5
		100	1.02 × 10^8^	97.1
		10	0.94 × 10^8^	89.5
		0	1.05 × 10^8^	100.0
	37 °C	1000	0.78 × 10^8^	104.0
		100	0.67 × 10^8^	89.3
		10	0.89 × 10^8^	118.8
		0	0.75 × 10^8^	100.0
BoHV-1 (4016)	4 °C	1000	5.6 × 10^6^	86.2
		0	6.5 × 10^6^	100.0
	22 °C	1000	5.5 × 10^6^	102.0
		0	5.4 × 10^6^	100.0
	37 °C	1000	2.8 × 10^6^	87.5
		0	3.2 × 10^6^	100.0

^a^ The residual infectivity in wells without of 2,5-DHBA–gelatin conjugate was taken as 100%.

### 2.6. Effect of 2,5-DHBA–Gelatin on the Attachment of Viruses to Target Cells

**Figure 4:**
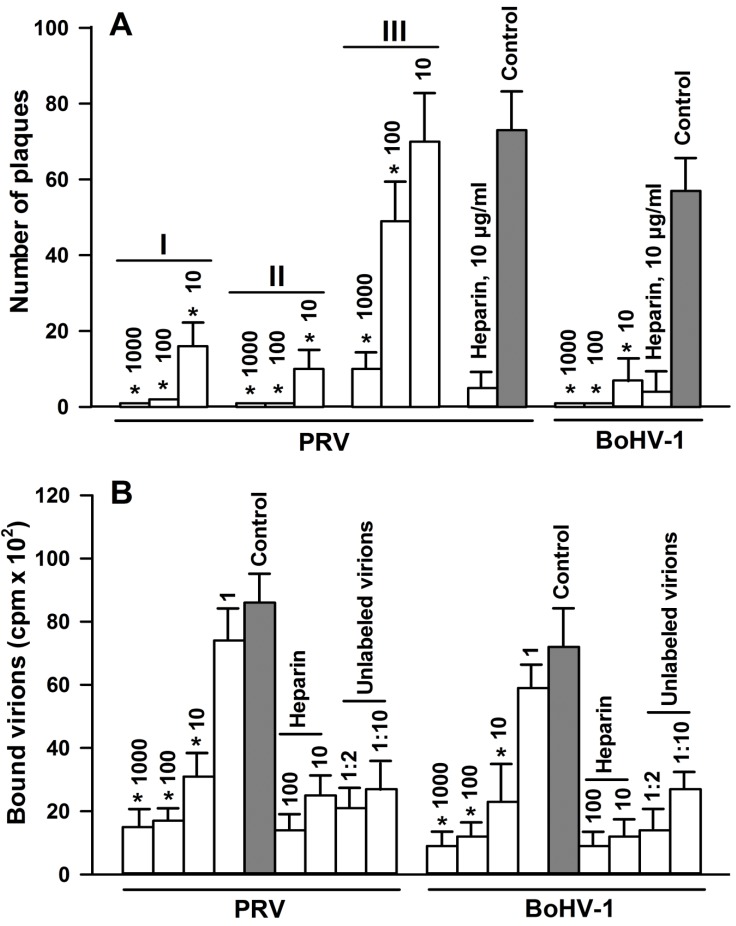
Effects of the 2,5-DHBA–gelatin conjugate on viral plaque formation (**A**) and on the attachment of radiolabeled PRV and BoHV-1 virions (**B**). (**A**) PRV was added to cells either simultaneously with the conjugate (I), or the conjugate was pre-incubated with cells for 1 h at 4 °C followed by the addition of PRV without the washing (II) or after the washing of cells with cold PBS (III). BoHV-1 was added simultaneously with the conjugate. The cells were incubated for 1 h at 4 °C and washed after which viral plaques were detected after incubation for 36–48 h at 37 °C; (**B**) Cell monolayers in 96-well plates were chilled and washed after which a constant amount of [^35^*S*]-labelled PRV and BoHV-1 virions (100,000-300,000 cpm/well) was incubated for 2 h at 4 °C in the presence of increasing concentrations of 2,5-DHBA–gelatin and heparin. To assess the specificity of binding of virions to cells, chilled and washed cells were incubated at 4 °C for 1 h with purified unlabeledvirions at different dilutions followed by incubation with radiolabeled virions (100,000–300,000 cpm/well) for 2 h at 4 °C. 2,5-DHBA–gelatin and heparin concentrations (µg/mL) and unlabelled purified virion dilutions are indicated above the bars. The amount of cell-bound radiolabeled virions was determined as described in Materials and Methods. Each value is the mean ± SD of three to four repeats. Asterisks indicate the statistical difference from the control: * *p* < 0.05.

To elucidate the effect of 2,5-DHBA–gelatin on the adsorption of the viruses to cells, BHK-21 and MDBK cells were chilled and incubated for 1 h with 2,5-DHBA–gelatin and appropriate viruses at 4 °C to exclude the penetration of viruses into cells during virus adsorption to cells. The cells were washed to remove the unbound virus and 2,5-DHBA–gelatin, cell monolayers were overlaid with semi-solid Dulbecco's modified Eagle's medium (DMEM) containing 0.5% methylcellulose (DMEM–methylcellulose), incubated for 36–48 h at 37 °C, and viral plaques were stained by the immunoperoxidase method. Each plaque corresponded to one viable virus particle adsorbed onto cells at low temperature. The 2,5-DHBA–gelatin conjugate strongly inhibited the adsorption of both alphaherpesviruses to cells in a dose-dependent manner (Figure 4A). As expected, the control substance, heparin, at a concentration of 5–10 µg/mL strongly inhibited the attachment of PRV and BoHV-1 to cells (Figure 4A). Since the inhibition of adsorption by the 2,5-DHBA–gelatin conjugate was comparable to the overall inhibition of virus infectivity observed in the end-point titration assay, this suggested that the inhibition of the attachment of the viruses to target cells significantly contributes to the overall antiviral activity of 2,5-DHBA–gelatin.

The 2,5-DHBA–gelatin conjugate can inhibit the attachment of virus to cells by interacting either with the viral or cellular proteins involved in the binding of the virus to cells. The interaction of the conjugate with viral proteins was examined in experiments aimed at determining the virucidal effects of 2,5-DHBA–gelatin (see above). We observed no decrease of titers of viruses in experiments when 2,5-DHBA–gelatin was incubated with the virus and further diluted 1000-fold before titration (Table 3). This suggests that, even if 2,5-DHBA–gelatin binds to virions, the binding is unstable and does not prevent the infection of cells by the viruses after dilution. In order to elucidate whether 2,5-DHBA–gelatin stably binds to cellular viral receptors, time-of-addition experiments were run using the PRV Ka strain. BHK-21 cells were preincubated at 4 °C with 2,5-DHBA–gelatin at different concentrations before the addition of the virus to allow 2,5-DHBA–gelatin binding to cellular receptors. Then, the virus was added either directly to wells without removing 2,5-DHBA–gelatin, or the cells were briefly washed three times with cold PBS before the virus was added. Then, cells were incubated for 1 h at 4 °C to allow the binding of virus to them, washed, and incubated at 37 °C for 36–48 h to determine the amount of viral plaques. As shown in Figure 4A,II, if the virus was added without removing 2,5-DHBA–gelatin, the adsorption of the virus to target cells was inhibited to a somewhat greater extent as compared to the experiments without the preincubation of 2,5-DHBA–gelatin with cells (Figure 4A,I). When 2,5-DHBA–gelatin was removed and cells were briefly washed, the inhibition of adsorption strongly decreased (Figure 4A,III). This suggests that no stable attachment of the 2,5-DHBA–gelatin conjugate to cell virus receptors occurs.

To further assess the influence of the 2,5-DHBA–gelatin conjugate on the direct binding of the viruses to cells, the effect of 2,5-DHBA–gelatin on the attachment of radio labeled PRV and BoHV-1 virions to target cells was determined. The results are presented in Figure 4B. The binding of radiolabeled PRV and BoHV-1 virions to cells was strongly inhibited by respective unlabeled virions, indicating the specificity of binding. The control substance, heparin, at concentrations of 10 and 100 μg/mL also strongly reduced the attachment of virions to cells. The 2,5-DHBA–gelatin polymer decreased the attachment of [^35^*S*]-labeled virions to cells in a dose-dependent manner. Unlabeled virions, heparin, and 2,5-DHBA–gelatin did not completely prevent the binding of [^35^*S*]-labeled virions to cells, probably due to the nonspecific binding of virions to cells. For both viruses, the 2,5-DHBA–gelatin concentration that causes a 50% inhibition of the binding of virions to cells was in the range of 1–10 µg/mL, *i.e.*, comparable to the *IC_50_* value obtained in the plaque reduction assay.

Together, these results indicated that 2,5-DHBA–gelatin inhibits the direct binding of PRV and BoHV-1 to cells, which results in a blocking of viral plaque formation. The 2,5-DHBA–gelatin polymer must be present in the medium during the adsorption of viruses to cells to impair the attachment of PRV and BoHV-1 to target cells.

### 2.7. Effect of 2,5-DHBA–Gelatin on Penetration, Cell-to-Cell Spread, and Production of Infectious Virus Particles

**Figure 5:**
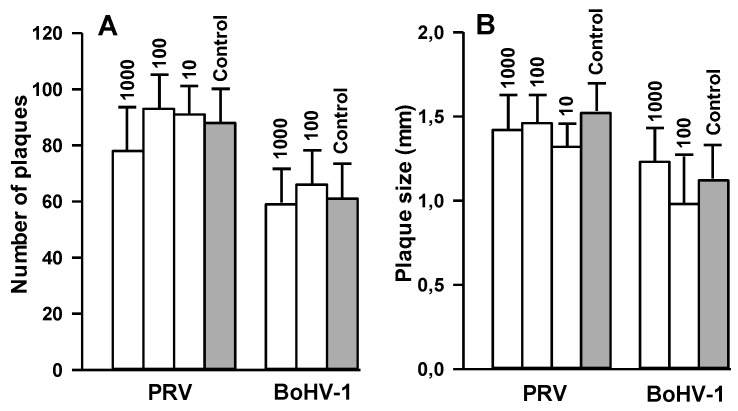
Effect of the 2,5-DHBA–gelatin conjugate on the penetration of viruses into cells (**A**) and the cell-to-cell spread of viruses (**B**). (**A**) The viruses were adsorbed to cells at 4 °C and washed following which 2,5-DHBA–gelatin was added to cells, and cells were incubated at 37 °C for 1 h to allow the viruses to penetrate into the cells. Unpenetrating viruses were inactivated, and viral plaques were detected after a 36–48-h incubation at 37 °C; (**B**) The viruses were adsorbed to cells, allowed to penetrate the cells, and unpenetrating viruses were inactivated as described in (**A**). Then, the cells were overlaid with DMEM-metylcellulose containing different concentrations of 2,5-DHBA–gelatin and incubated for 48–72 h at 37 °C, after which viral plaques were stained, and viral plaque sizes were determined. 2,5-DHBA–gelatin concentrations (µg/mL) are indicated above bars. Each value is the mean ± SD of three to five repetitions (**A**) or no less than 50 (**B**) repetitions.

Then, we tested whether 2,5-DHBA–gelatin can inhibit the penetration of the viruses into cells. The viruses were adsorbed to cells under conditions that prevent the penetration of viruses into them (4 °C). Then the viruses were allowed to penetrate into cells in the presence or in the absence of 2,5-DHBA–gelatin, and unpenetrating viruses were inactivated. After that, cells were overlaid with DMEM-methylcellulose and incubated for 36–48 h at 37 °C, after which the amount of viral plaques was determined. Each plaque corresponds to one virus particle that penetrates into the cell during the incubation with or without 2,5-DHBA–gelatin. The results presented in Figure 5A indicated that 2,5-DHBA–gelatin did not inhibit the penetration of PRV and BoHV-1 into cells.

We also examined the effect of 2,5-DHBA–gelatin on cell-to-cell spread. The viruses were adsorbed to cells and allowed to penetrate into cells, and unpenetrating viruses were inactivated. The cells were overlaid with DMEM-methylcellulose containing serial dilutions of 2,5-DHBA–gelatin and incubated at 37 °C for 48 h (for PRV) or 72 h (for BoHV-1). Viral plaques were stained, and plaque sizes reflecting the cell-to-cell spread of viruses were determined. The results are presented in Figure 5B. The 2,5-DHBA–gelatin conjugate had no inhibitory effect on PRV and BoHV-1 cell-to-cell spread.

**Figure 6:**
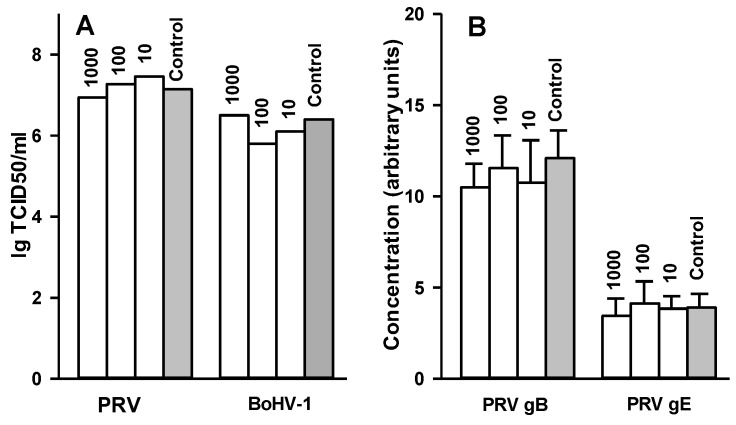
Effects of 2,5-DHBA–gelatin conjugate on production of infectious PRV and BoHV-1 particles (**A**) and expression of PRV glycoproteins B and E (**B**). The viruses were adsorbed on appropriate cells at 4 °C, incubated for 1 h at 37 °C to allow the virus to penetrate cells, and unpenetrating viruses were inactivated. The cells were incubated in DMEM containing different concentrations of 2,5-DHBA–gelatin for 10 h at 37 °C. Then, virus titres (**A**) and relative concentrations of PRV glycoproteins B and E (**B**) were determined in cell lysates. The relative concentrations of PRV gB and gE (**B**) are presented in arbitrary units as the mean ± SD of four repetitions. 2,5-DHBA–gelatin concentrations (µg/mL) are indicated above bars.

Finally, we tested whether 2,5-DHBA–gelatin inhibited the production of infectious virus particles and the expression of two PRV glycoproteins (gB and gE) after the penetration of the viruses into the cell. The viruses were adsorbed to cells at a multiplicity of 3–5 pfu per cell. The adsorbed viruses were allowed to penetrate into cells, and unpenetrating viruses were inactivated. The cells were overlaid with a medium containing different dilutions of 2,5-DHBA–gelatin and incubated for 10 h at 37 °C to allow the production of the viruses in cells. Then, virus titers and relative concentrations of virus glycoproteins B and E (for PRV experiments) were determined in cell lysates. The results are presented in Figure 6A,B. The 2,5-DHBA–gelatin conjugate had no inhibitory effect on the production of infectious virus particles in cells and the expression of PRV glycoproteins B and E.

## 3. Discussion

Numerous negatively charged polymers exhibit potent antiviral activities against various enveloped viruses (for review, see reference [1]). We synthesized a 2,5-DHBA–gelatin conjugate by the laccase-mediated oxidation of 2,5-DHBA in the presence of gelatin and evaluated its antiviral activity against two alphaherpesviruses, PRV and BoHV-1. Gelatin, which results from a partial degradation of water-insoluble collagen has been widely used in food and pharmaceutical industries. Due to low immunogenicity and cytotoxicity and good biodegradability, gelatin is used in the preparation of various biomaterials, including matrices for drug carriers [47]. Since it has phenolic groups, 2,5-DHBA was chosen as a substance for covalent conjugation to gelatin; therefore, it can be oxidized by laccase; moreover, it contains a carboxyl group, which bears a negative charge.

The polymerization of phenols by laccase is a free radical process [48]. During the conjugation to gelatin, radicals of phenolic compounds interact with residues in the side chains of gelatin to form covalent bonds [49]. We optimized the conjugation of 2,5-DHBA to gelatin and determined the optimal concentrations of the reactants (2,5-DHBA, gelatin, and laccase), which allowed us to synthesize the water-soluble 2,5-DHBA–gelatin conjugate with the yield of 70%–80%. The conjugate was soluble at alkaline, neutral, and weakly acidic pH values (Figure 3A), while at pH values below 3.0 it precipitated, probably due to the protonation of the free carboxyl groups of 2,5-DHBA, for which the pK value is 2.9. A gel filtration analysis showed a rather high heterogeneity of the 2,5-DHBA–gelatin conjugate: a major broad peak with the estimated apparent molecular weight maximum of about 175 kDa and a minor peak corresponding to a small fraction with a molecular weight of approximately 35 kDa were seen on the chromatogram (Figure 3B). The conjugate had an absorption maximum at 320 nm (Figure 2A), due to the presence of 2,5-DHBA chromophore bound to gelatin. The absorption intensity decreased with increasing wavelength, as is evidenced from the UV-Vis spectrum (Figure 2A). Similar spectra are characteristic of polymers with unsaturated conjugated bonds and aromatic rings, such as lignin and humic acids [50,51], which might indicate the presence of such types of bonds in 2,5-DHBA–gelatin. The FT-IR spectra of 2,5-DHBA–gelatin conjugate confirmed the formation of covalent bonds between 2,5-DHBA and gelatin. Not all 2,5-DHBA functional groups were shown to be involved in the formation of bonds with gelatin.

The 2,5-DHBA–gelatin conjugate showed no direct toxicity to three cell lines: Vero, BHK-21, and MDBC and did not inhibit cell growth at a concentration of 1000 µg/mL (the highest concentration tested). It is noteworthy that the toxicity of natural and synthetic polymers is often relatively low, due to the inability of polymers to penetrate the cells [1,2,7,10,14,17].

The 2,5-DHBA–gelatin conjugate showed a strong antiviral activity against two PRV strains (the laboratory strain Ka and the vaccine strain Bartha K61) and BoHV-1 virulent strain 4016. The *IC*_50_ values for the PRV strains Ka and Bartha K61 were 1.5–5.5 µg/mL and 8.3–15.1 µg/mL, respectively. For the BoHV-1 strain 4016, the *IC*_50_ values were 0.5–0.7 µg/mL (Table 2). It is noteworthy that different natural and synthetic polymers with antiviral activity were reported to have comparable *IC*_50_ concentrations against different enveloped viruses [1,4,5,6,7,10,11,12,14,15,16,17]. We observed pronounced differences in the antiviral activity of the 2,5-DHBA–gelatin conjugate against two PRV strains. Conjugate preparation No 4 was more active against PRV strain Ka (*IC*_50_ 2.4–2.8 µg/mL) than against PRV vaccine strain Bartha K61 (*IC*_50_ 8.5–10 µg/mL). It is known that the results of the antiviral tests of polymers can differ depending on virus strains, cell types and experimental conditions [52]. The less pronounced effect of the conjugate on PRV strain Bartha K61 is probably due to the relatively poor presentation of glycoprotein C in the virions of this virus strain [53], which may results in a weaker HSPG-dependent attachment of Bartha K61 virions to cell surface and, as a result, in a lower sensitivity of the virus to the substances that impair the attachment of virus to cell surface HSPGs.

The 2,5-DHBA–gelatin conjugate strongly inhibited the adsorption of PRV and BoHV-1 to target cells but had no direct virucidal effect on the viruses and did not affect their penetration into cells, cell-to-cell spread, and production of infectious virus particles in cells. It is noteworthy that we observed comparable antiviral activity against PRV (Ka strain) for high- and low-molecular-weight fractions of the polymer (150–200 kDa and 25–50 kDa, respectively). Both fractions of the 2,5-DHBA–gelatin conjugate inhibited the attachment of the virus to cells (data not shown). The results obtained are in agreement with the data indicating that numerous natural and synthetic polymers (heparin, lignins, sulfated polysaccharides and lignins, DHP, and others) impair the attachment of various enveloped viruses to cells [1,17]. This suggests that the molecular mechanisms of the antiviral activity of the 2,5-DHBA–gelatin polymer, heparins, lignins, lignin-like polymers may be quite similar. These natural and synthetic polymers are structurally different. Most probably, the electrostatic surface of these polymers has a similarity with that of heparin sulfate of cellular HSPGs, and the polymers compete with cell-surface HSPGs for binding to glycoproteins gC and/or gB of alphaherpesviruses.

Our results suggest that the 2,5-DHBA–gelatin conjugate is a promising polymer for developing antiviral formulations, in particular formulations for topical use, to be used against alphaherpesvirus infections. 2,5-DHBA–gelatin hybrid polymer can be rather easily synthesized using enzymatic oxidative coupling. Owing to a significant homology between the members of the *Alphaherpesvirinae* and similarity of mechanisms of entry of different alphaherpesviruses into cells, it can be expected that the 2,5-DHBA–gelatin hybrid polymer would also inhibit the entry of other alphaherpesviruses into cells as well as the entry of other enveloped viruses, for example, HIV-1, hepatitis C virus, and Kaposi’s sarcoma-associated herpes virus that utilize HSPGs to gain entry into cells.

## 4. Materials and Methods

### 4.1. Materials and Chemicals

Cell culture flasks, 96- and 48-well TC plates, and Petri dishes were from Corning Inc. (New York, NY, USA). Sterilizing filters (0.22 µm) were purchased from Millipore (Billerica, MA, USA). Vivaspin 2 centrifugal concentrators were from Vivaproducts, Inc. (Littleton, CO, USA). Fetal Bovine Serum was purchased from GE Healthcare (Little Chalfont, UK). Gelatin from porcine skin (48722 Fluka, Germany) was used in the experiments. All other chemicals were of analytical grade and were obtained from Sigma-Aldrich Corporation (St. Louis, MO, USA). The preparation and characterization of monoclonal antibodies (MAbs) against PRV glycoproteins B and E have been described [54,55]. Home prepared BoHV-1-specific MAb 6-1 was used for the immunoperoxidase detection of BoHV-1 plaques.

### 4.2. Cells and Viruses

The PRV strains (the laboratory strain Ka and the vaccine strain Bartha K61) and BoHV-1 (the virulent strain 4016) were obtained from the collection of viruses of the Institute of Veterinary Medicine (Kiev, Ukraine). Baby hamster kidney (BHK-21) cells and African green monkey kidney (Vero) cells were used for the cultivation of PRV, and Madin-Darby Bovine Kidney (MDBK) cells were used for the propagation of BoHV-1. Cell lines were from the Cell Culture Collection of Vertebrates (St. Petersburg, Russia). Cells were grown in DMEM supplemented with 10% FCS (DMEM/FCS) and antibiotics (100 IU/mL penicillin, 100 µg/mL streptomycin) and were cultured in cell culture flasks at 37 °C in an atmosphere of 100% humidity and 5% CO_2_.

For the production of viruses, BHK-21 and MDBK cells were infected with the PRV (Ka strain) and BoHV-1, respectively, at a multiplicity of 1–3 PFU per cell. After 48 h, the virus-containing fluid was harvested and clarified by centrifugation at 5000 *g* for 10 min. Virions were purified from the clarified virus-containing fluid by sedimentation in a linear sucrose gradient [56]. For the preparation of radiolabeled virions, cells were infected as described above. After four hours, infected cells were thoroughly washed with methionine-free DMEM. Then methionine-free DMEM containing 1% FBS and 20 μCi of L-[^35^S]-methionine per mL of medium was added to cells. Forty eight hours later, the virus-containing fluid was harvested, and [^35^*S*]-methionine-labeled virions were purified as described above.

### 4.3. The Synthesis of the 2,5-DHBA–Gelatin Conjugate

First, 2,5-DHBA was conjugated with gelatin by laccase-catalyzed oxidation of 2,5-DHBA in the presence of gelatin. Laccase was purified from the culture of the fungus *Cerrena unicolor* VKM F-3196 as described earlier [57]. The laccase activity unit was defined as the amount of laccase that catalyzes the oxidation of 1 μM of 2,2′-azino-bis(3-ethylbenzothiazoline-6-sulfonic acid) (ε_420_ = 36,000 M^−1^ × cm^−1^) per minute at 30 °C. The laccase-catalyzed synthesis of the 2,5-DHBA–gelatin conjugate was run in a 50 mM Na-acetic buffer, pH 5.0. The reaction was started by addition of laccase to a solution of 2,5-DHBA and gelatin, and the mixture was kept at 30 °C under stirring at 200 rpm on a rotary shaker for 15 h. The concentrations of the reactants (2,5-DHBA, gelatin, and laccase) are given in the text. The reaction mixture was heated for 10 min in a boiling water bath to stop the reaction and dialyzed (molecular weight cut-off (MWCO), 10 kDa) against distilled water. The precipitate formed during the synthesis was separated by centrifugation at 5000 *g* for 30 min. The conjugate was concentrated using a Vivaspin 2 centrifugal concentrator (MWCO, 10 kDa).

### 4.4. Characterization of the 2,5-DHBA–Gelatin Conjugate

The synthesis of the conjugate was optimized using gel filtration of the reaction mixture on a 50 × 1.5 cm column packed with Sephadex G-75 for the control of resulting polymers. Elution was carried out with 0.1% NaOH at a flow rate of 0.5 mL/min. The molecular weight of the conjugate was determined by gel filtration on a HiLoad 26/60 Superdex 200 column calibrated with a gel filtration molecular weight marker kit 29,000–700,000 kDa (Sigma).

The UV-visible (UV-Vis) absorption spectrum of the conjugate was recorded in water at room temperature using a Shimadzu UV-1650 PC spectrophotometer. The study of the conjugate by Fourier transform infrared (FT-IR) spectroscopy was performed on an Agilent Cary 630 (Agilent Technologies) spectrometer within the range of 4000–650 cm^−1^ in the mode of attenuated total reflection.

To study the solubility of the conjugate at different pH values, samples of the conjugate dissolved in water at a concentration of 2 mg/mL were dialyzed against 50 mM universal Britton-Robinson buffer with different pH values. After the dialysis, the turbidity of the solution was measured at 600 nm.

### 4.5. Determination of In Vitro Cytotoxicity and Antiproliferative Activity of 2,5-DHBA–Gelatin Conjugate

The *in vitro* cytotoxicity and antiproliferative activity of 2,5-DHBA–gelatin for BHK-21, Vero, and MDBK cells were determined using the MTT assay. For the determination of cytotoxicity, the cells were seeded in DMEM/FCS in wells of 96-well plates (2.0–3.0 × 10^4^ cells per well) and were grown overnight till confluence. The medium was discarded, and 2,5-DHBA–gelatin diluted at different concentrations in DMEM/FCS or DMEM/FCS (control) were added to confluent monolayers of cells and incubated for 72 h. After incubation, 50 µL of MTT solution (5 mg/mL) was added to each well, and the plates were incubated at 37 °C for 2 h. The medium was carefully removed from the wells, and 200 µL of DMSO was added to each well and pipetted to dissolve crystals. The absorbance was measured at 550 nm, and the percentage of cytotoxicity was calculated relative to the control without the conjugate.

To determine the antiproliferative activity of the conjugate, the cells were plated in DMEM/FCS in wells of 96-well (5.0–7.0 × 10^3^ cells per well). Simultaneously with cells, 2,5-DHBA–gelatin diluted at different concentrations in DMEM/FCS or DMEM/FCS (control) was added to the wells, and the cells were incubated for 48 h. MTT staining of cells was performed as described above, and the concentration at which a 50% inhibition of cell growth occurs (*CC*_50_) was calculated.

### 4.6. Virus End-Point Titration Assay

BHK-21, Vero, and MDBK cells were grown in 96-well plates till confluence. The medium was changed for DMEM containing 1% FCS and different concentrations of 2,5-DHBA–gelatin. Thereafter, the viruses were titrated 10-fold on appropriate cells in rows with different concentrations of 2,5-DHBA–gelatin and in control rows without the conjugate. The plates were incubated for 48 h, and the cells were washed with PBS and fixed with cold ethanol. Then, PRV and BoHV-1 virus plaques were detected by the immunoperoxidase assay using the peroxidase conjugate of PRV-specific MAb 34/2 and BoHV-1-specific MAb 6-1. Each concentration of 2,5-DHBA–gelatin was analyzed in five to six repeats. A 50% tissue culture infectious dose (*TCID*_50_) was calculated by the Reed–Muench method [58].

### 4.7. Plaque Reduction Assay

BHK-21, Vero, and MDBK cells were grown in 24-well plates till confluence. Cell monolayers were then infected with 100–120 pfu of PRV or BoHV-1 in the absence or presence of 2,5-DHBA–gelatin at different concentrations and incubated for a further 1 h at 37 °C. After 1 h of adsorption, the cell monolayers were washed with PBS and overlaid with 0.5% methylcellulose in DMEM/1% FBS containing the 2,5-DHBA–gelatin conjugate at different concentrations. Each concentration of the 2,5-DHBA–gelatin conjugate was analyzed in five to six repeats. The plates were incubated for 48 h at 37 °C, virus plaques were stained as described above and counted under a microscope. The minimal concentration of the 2,5-DHBA–gelatin conjugate required to reduce the virus plaques number by 50% (*IC_50_*), was calculated by the regression analysis of the dose–response curves generated from these data.

### 4.8. Virus Adsorption Assay

Confluent monolayers of BHK-21 and MDBK cells grown in 48-well plates were chilled to 4 °C and washed three times with ice-cold PBS. Ice-cold samples of DMEM containing different concentrations of 2,5-DHBA–gelatin (250 µL) and PRV or BoHV-1 viruses (50–100 pfu in 250 µL) were added to wells with BHK-21 and MDBK cells, respectively. The plates were incubated for 1 h at 4 °C, washed three times with ice-cold PBS to remove unbound viruses and 2,5-DHBA–gelatin, and the cell monolayers were covered with 0.5% methylcellulose in DMEM/1% FBS. The plates were incubated at 37 °C for 36–48 h. Then, the cells were fixed with cold ethanol, and viral plaques were detected by the immunoperoxidase assay using the peroxidase conjugate of PRV- and BoHV-1-directed MAbs and counted under an inverted microscope.

In some experiments with PRV strain Ka, BHK-21 cells were preincubated for 2 h at 4 °C with different concentrations of 2,5-DHBA–gelatin diluted in DMEM before the addition of viruses. Then, the virus (50–100 pfu) was added either directly to wells without removing 2,5-DHBA–gelatin, or the cells were briefly washed three times with cold PBS before addition of 50–100 pfu of the virus. The plates were incubated for 1 h at 4 °C followed by three washes with ice-cold PBS and addition of 0.5% methylcellulose in DMEM/1% FBS. Then, the plates were incubated for 36 h at 37 °C, and viral plaques were detected by the immunoperoxidase assay and counted as described above.

### 4.9. Binding of Radiolabeled Virions to Cells

Confluent BHK-21 or MDBK cell monolayers grown in 96-well plates were chilled to 4 °C. After the removal of the medium, cells were washed three times with cold PBS and incubated for 2 h at 4 °C with radiolabeled virions (100,000–300,000 cpm/well) diluted in DMEM containing 1% BSA (DMEM–BSA) and increasing concentrations of 2,5-DHBA–gelatin or heparin. The cells were washed with cold PBS, lysed in PBS containing 1% SDS for 1 h at 37 °C, and the cell-associated radioactivity was determined by liquid scintillation counting. Each experiment was performed in triplicate.

To assess the specificity of the binding of PRV and BoHV-1 virions to cells, chilled and washed BHK-21 and MDBK cell monolayers in 96-well plates were incubated at 4 °C for 1 h with purified unlabeledvirions at different dilutions. After this, the cells were washed with cold PBS and incubated with a constant amount of radiolabeled virions (100,000–300,000 cpm/well) for 2 h at 4 °C. Cell monolayers were washed with cold PBS and lysed after which the cell-bound radioactivity was counted. Each experiment was performed in triplicate.

### 4.10. Virus Penetration Assay

BHK-21 and MDBK cell monolayers in 48-well plates were chilled to 4 °C. After the removal of the medium, cells were washed three times with ice-cold PBS. BHK-21 and MDBK cell were incubated for 1 h at 4 °C with PRV and BoHV-1 (approximately 100–200 pfu/well), respectively. After washing three times with ice-cold PBS, cell monolayers were overlaid with the medium containing different concentrations of the 2,5-DHBA–gelatin conjugate and incubated for 1 h at 37 °C to allow the penetration of the cell-absorbed virus into cells. To inactivate unpenetrating viruses, the cells were treated with citrate buffer (40 mM citric acid, 10 mMKCl, 135 mMNaCl, pH 3.0) for 1 min followed by three washes with DMEM. The cells were overlaid with 0.5% methylcellulose in DMEM/1% FBS. Then, the plates were incubated at 37 °C for 36–48 h, and viral plaques were detected by the immunoperoxidase assay and counted as described above.

### 4.11. Influence of 2,5-DHBA–Gelatin on Cell-to-Cell Spread of PRV and BoHV-1

BHK-21 and MDBK cells were exposed to PRV and BoHV-1, respectively, the viruses were allowed to penetrate into cells, and unpenetrating viruses were inactivated as described above. The cells were overlaid with 0.5% methylcellulose in DMEM/1% FBS containing serial dilutions of 2,5-DHBA–gelatin and neutralizing PRV- and BoHV-1-specific MAbs to exclude the virus spread via free viruses. The plates were incubated at 37 °C for 48 h (for PRV detection) or for 72 h (for BoHV-1 detection), and viral plaques were detected by the immunoperoxidase assay as described above. Photos of arbitrarily chosen fields of vision with virus plaques were made, and plaque sizes were determined. At least 50 viral plaques were analyzed for each experiment.

### 4.12. Direct Virucidal Effect of 2,5-DHBA–Gelatin on Viruses

PRV and BoHV-1 (10^6^–10^7^pfu/mL) were treated with equal volumes of serial dilutions of 2,5-DHBA–gelatin for 1 h at 4, 22 and 37 °C. The viruses were diluted 1000-fold in DMEM, titrated in 96-well plates, and the virus titers were determined as described above.

### 4.13. Influence of 2,5-DHBA–Gelatin on Production of Infectious PRV and BoHV-1 Particles and Expression of PRV Glycoproteins B and E

BHK-21 and MDBK cells were exposed to PRV and BoHV-1, respectively, (approximately 3–5 pfu/cell), the viruses were allowed to penetrate into cells, and unpenetrating viruses were inactivated as described above. The cells were overlaid with DMEM/1% FBS containing serial dilutions of 2,5-DHBA–gelatin. The plates were incubated for 10 h at 37 °C. The cells with the medium were subjected to two freeze/thaw cycles followed by the determination of virus titers as described above. To determine the influence of 2,5-DHBA–gelatin on the expression of PRV glycoproteins B and E, cellswere lyzed with 1% Triton X-100. The relative concentration of PRV glycoproteins B and E was measured using two-site “sandwich” gB-ELISA and gE-ELISA [59] and expressed in arbitrary units.

### 4.14. Statistical Analysis

Each experiment was performed independently at least three times, and representative results are shown in the tables and figures. The data are expressed as the mean ± SD. The statistical significance was determined by the Student’s *t*-test, and *p* < 0.05 was considered significant.
